# Identification of berberine as a potential therapeutic strategy for kidney clear cell carcinoma and COVID-19 based on analysis of large-scale datasets

**DOI:** 10.3389/fimmu.2023.1038651

**Published:** 2023-03-23

**Authors:** Zhihua Zheng, Xiushen Li, Kechao Nie, Xiaoyu Wang, Wencong Liang, Fuxia Yang, Kairi Zheng, Yihou Zheng

**Affiliations:** ^1^ Department of Nephrology, Shenzhen Traditional Chinese Medicine Hospital, The Fourth Clinical Medical College of Guangzhou University of Chinese Medicine, Shenzhen, Guangdong, China; ^2^ Shenzhen Key Laboratory, Shenzhen University General Hospital, Shenzhen, Guangdong, China; ^3^ Department of Gastroenterology, The Second Xiangya Hospital of Central South University, Changsha, Hunan, China; ^4^ Department of Nephrology, Health College of Guangdong Pharmaceutical University, Guangzhou, Guangdong, China; ^5^ Traditional Chinese Medicine Department, Peking University Shenzhen Hospital, Shenzhen, Guangdong, China

**Keywords:** coronavirus disease 2019, kidney clear cell carcinoma, berberine, immune mechanism, molecular docking

## Abstract

**Background:**

Regarding the global coronavirus disease 2019 (COVID)-19 pandemic, kidney clear cell carcinoma (KIRC) has acquired a higher infection probability and may induce fatal complications and death following COVID-19 infection. However, effective treatment strategies remain unavailable. Berberine exhibits significant antiviral and antitumour effects. Thus, this study aimed to provide a promising and reliable therapeutic strategy for clinical decision-making by exploring the therapeutic mechanism of berberine against KIRC/COVID-19.

**Methods:**

Based on large-scale data analysis, the target genes, clinical risk, and immune and pharmacological mechanisms of berberine against KIRC/COVID-19 were systematically investigated.

**Results:**

In total, 1,038 and 12,992 differentially expressed genes (DEGs) of COVID-19 and KIRC, respectively, were verified from Gene Expression Omnibus and The Cancer Genome Atlas databases, respectively, and 489 berberine target genes were obtained from official websites. After intersecting, 26 genes were considered potential berberine therapeutic targets for KIRC/COVID-19. Berberine mechanism of action against KIRC/COVID-19 was revealed by protein-protein interaction, gene ontology, and Kyoto Encyclopedia of Genes and Genomes with terms including protein interaction, cell proliferation, viral carcinogenesis, and the PI3K/Akt signalling pathway. In COVID-19 patients, ACOX1, LRRK2, MMP8, SLC1A3, CPT1A, H2AC11, H4C8, and SLC1A3 were closely related to disease severity, and the general survival of KIRC patients was closely related to ACOX1, APP, CPT1A, PLK1, and TYMS. Additionally, the risk signature accurately and sensitively depicted the overall survival and patient survival status for KIRC. Numerous neutrophils were enriched in the immune system of COVID-19 patients, and the lives of KIRC patients were endangered due to significant immune cell infiltration. Molecular docking studies indicated that berberine binds strongly to target proteins.

**Conclusion:**

This study demonstrated berberine as a potential treatment option in pharmacological, immunological, and clinical practice. Moreover, its therapeutic effects may provide potential and reliable treatment options for patients with KIRC/COVID-19.

## 1 Introduction

Since its discovery in December 2019, severe acute respiratory syndrome coronavirus 2 (SARS-CoV-2) has spread and levied significant damage to the economies, quality of life, and psychological health of individuals. As of 11 August 2022, the World Health Organization (WHO) (https://covid19.who.int/) reported a 24-hour-emergence of 866,828 new COVID-19 cases, a 580 million increase in the total number of COVID-19 patients worldwide and 6.4 million COVID-19-induced deaths. Despite the rapid development of vaccines and population-wide immunisation campaigns (1, 2), the global epidemiological scenario remains critical owing to significant infectivity, SARS-CoV-2 mutations, and declining neutralising antibody titers (3–6). Given that 5% of COVID-19 patients with acute respiratory distress syndrome (ARDS) require mechanical ventilation and intensive care unit (ICU) admittance (7), effective treatment approaches are urgently required to alleviate symptoms and reduce mortality.

Cancer and COVID-19 may be related as COVID-19 infection is more common in cancer patients (8, 9). In addition, COVID-19 patients with cancer are at a greater risk for serious complications and unfavourable prognosis than those without cancer (10–12), particularly immunocompromised patients (13). Most renal cell carcinoma (kidney clear cell carcinoma, KIRC) cases are malignant with significant mortality (14). Following COVID-19 infection, 28.5% and 19.3% of patients experience immediate renal impairment and die, respectively, indicating that the infection quickly attacks the kidney and endangers the lives of patients (15, 16). This may be linked to the development of sickle cell traits and enhanced ACE2 expression (17, 18). Furthermore, following COVID-19 infection, KIRC patients experience severe complications and elevated mortality risk (19). Similarly, the COVID-19 pandemic delays medical care for KIRC patients (20).KIRC patients have a higher risk of infection and may develop fatal complications and higher mortality from COVID-19.

**Figure 1:**
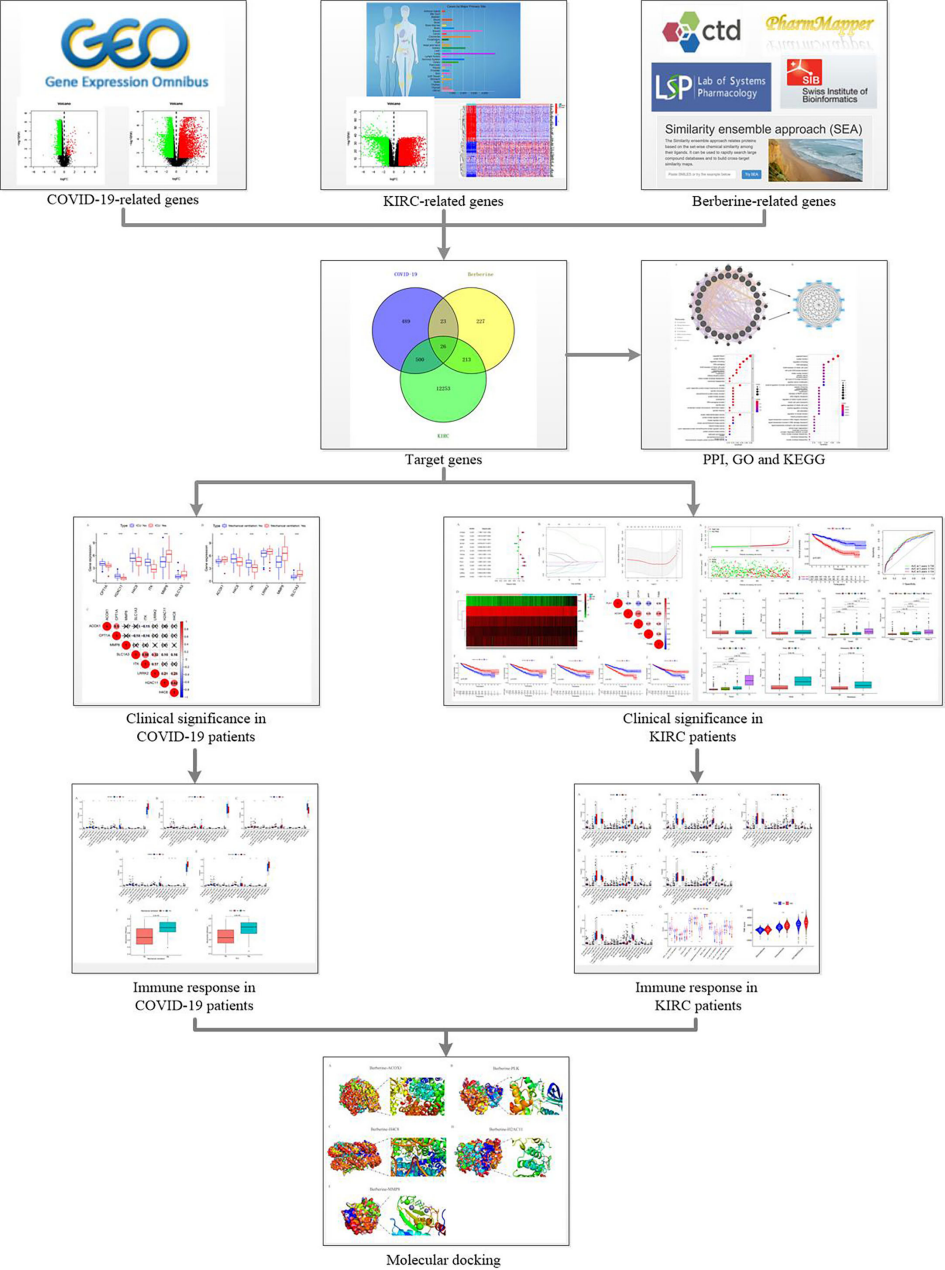
Flow chart.

Therefore, we sought effective treatment methods to reduce the progressive COVID-19-induced deterioration and improve the survival of patients with KIRC. Berberine is a natural compound with strong antibacterial, antiviral, and antitumour activities (21–27). It can resist the inflammatory response of acute lung injury by facilitating nuclear translocation and phosphorylation of nuclear factor erythroid 2–related factor 2 (Nrf2) to reduce the generation of inflammatory factors and reactive oxygen species (ROS) (28); berberine induced apoptosis in virus-infected cells by promoting ROS production (29, 30). This dual effect of resistance to disease damage and viral destruction is specific and important; by directly interacting with the virion, berberine counteracts the infectiousness and inhibits SARS-CoV-2 replication (31). Moreover, berberine decreases circulating inflammatory mediators, including interleukin (IL)-6, tumour necrosis factor (TNF)-α, and C-reactive protein (CRP), in patients with severe COVID-19 (32). Additionally, in combination with photodynamic therapy, berberine accelerates autophagy and apoptosis of KIRC cells by promoting ROS generation, resulting in KIRC cell death (33). It also strengthens apoptosis in KIRC cells by stimulating ROS generation by decreasing c-FLIP and Mcl-1 protein regulation (34). Hence, berberine can inhibit SARS-CoV-2 infection and reproduction, reduce the inflammatory response in COVID-19 patients, and promote apoptosis of KIRC cells by facilitating ROS generation. This study aimed to further explore the berberine therapeutic mechanism in treating KIRC/COVID-19 patients to provide treatment evidence and strategy for these patients (**Figure 1**).

## 2 Materials and methods

### 2.1 Identification of KIRC/COVID-19-related genes and obtainment of berberine target genes

The COVID-19-related transcriptomic RNA-sequencing datasets, GSE157103 and GSE171110, were acquired from GEO datasets (https://www.ncbi.nlm.nih.gov/geo/). By using the “limma” package in R software (version 4.2) with the the filtering criterion of *p* < 0.05, and | log fold change | of > 0.585, the COVID-19 differentially expressed genes (DEGs) in two datasets were verified. DEGs of two datasets were intersected reciprocally to screen out the most credible COVID-19- related genes.

The KIRC RNA-seq dataset was downloaded from The Cancer Genome Atlas (TCGA, https://portal.gdc.cancer.gov/repository). ​The analogous calculation and filtering criterion were carried out for the identification of KIRC-related genes.

To discover the berberine therapeutic target gene, we deploy a comprehensive retrieval strategy *via* official websites, including CTD (http://ctdbase.org/), Parm Mapper (http://www.lilab-ecust.cn/pharmmapper/), SEA (https://sea.bkslab.org/), Swiss Target Prediction (http://www.swisstargetprediction.ch/) and TCMSP (http://tcmspw.com/tcmsp.php/). These genes from various websites were merged and considered berberine target genes.

Ultimately, the COVID-19- related genes, KIRC-related genes and berberine target genes were intersected. And these overlapping genes represented therapeutic target genes of berberine against KIRC/COVID-19.

### 2.2 Functional analyses of therapeutic target genes of berberine against KIRC/COVID-19

To determine the interactions between target genes of berberine against KIRC/COVID-19, the GeneMANIA (http://genemania.org/), a website predicting the interactions between genes based on the label propagation algorithm and linear regression-based algorithm, was performed to construct a protein-protein interaction (PPI) network. PPI network data was imported into Cytoscape software to build the core network based on crucial topological parameters, including Betweenness-Centrality, Closeness-Centrality and Degree.

In addition, Gene Ontology (GO) and Kyoto Encyclopedia of Genes and Genomes (KEGG) enrichment analysis were executed *via* the R package “clusterProfiler”, making clear the underlying therapeutic mechanism of berberine against KIRC/COVID-19.

### 2.3 Exploring the clinical significance of target genes in COVID-19 patients

​ Due to the lack of clinical information in the GSE171110 dataset, the GSE157103 dataset was selected as the primary clinical research objective. Admittedly, mechanical ventilation is an essential life support therapy for patients with respiratory distress, and most critically ill COVID-19 patients desperately need mechanical ventilation (35, 36), which is an important clinical indicator of the severity of COVID-19 patients, especially in the respiratory system. Whether admission in ICU is also a key clinical information to judge the condition of patients with COVID-19, which pays greater attention to holistic life support therapy. Then, the Wilcoxon test was performed to examine which genes are associated with clinical factors of patients with COVID-19. After screening, the target genes were identified the association with each other *via* the Spearman correlation test.

### 2.4 Exploring the clinical significance of target genes in KIRC patients

Based on the “survival”, “survminer” and “glmnet” package in R software, the target genes of KIRC patients were identified by using the univariate COX regression analysis and LASSO Cox regression analysis successively. Genes after being screened would be divided equally into high- and low- expression groups. Kaplan–Meier survival analysis was applied to analyze which genes are closely related to overall survival (OS) between the high- and low- expression groups. These genes would be calculated as risk score according to the computational formula: risk score = sum of coefficients × gene expression level. The KIRC patients were divided into low- and high-risk groups based on the median of risk score. The heatmap could visually describe the difference of gene expression between low- and high-risk groups, and the correlation of each gene detected by the Spearman correlation test.

### 2.5 Verification between risk and clinical information in KIRC patients

The relationship between risk signature and OS was proved using Kaplan–Meier survival analysis. The risk signature’s sensitivity and specificity were further evaluated through the area under the curve (AUC) in the time‐dependent receiver operating characteristic (ROC) analyses performed by R packages “survivalROC”. The clinical information, including age, gender, grade, tumour stage, tumour, node and metastasis, were equipped to describe the survival state of KIRC patients in multidimensional and depth. And the differences in the risk signature at different clinical stages would be compared mutually.

### 2.6 Revelation of potential immune mechanism in KIRC/COVID-19

Immune response plays an irreplaceable role in the development and treatment of KIRC (37, 38) and COVID-19 infection (39, 40). We estimated 22 immune cell infiltration in KIRC/COVID-19 patients respectively *via* R package “CIBERSORT”, and analyzed the difference of each immune cell infiltration in genes of berberine against KIRC/COVID-19. Moreover, analysis of immunological function was further investigated through R package “GSVA” and “GSEABase”. The infiltration of stromal cells and immune cells was assessed by R software package (“estimate”), for making clear the tumour microenvironment (TME).

### 2.7 Molecular docking

Molecular docking analysis was carried out to predict predominant binding modes of berberine to KIRC/COVID-19-related proteins. We collected structures of target proteins and berberine from RCSB PDB database (https://www.rcsb.org/) and PubChem (https://pubchem.ncbi.nlm.nih.gov/). After removing redundant ligand and hydrone of target proteins *via* Pymol software (version 2.4), we loaded structures of proteins and berberine into Autodock Vina to perform the molecular docking. When the docking affinity scores < -5.0 kcal/mol, it indicates that berberine has strong binding interaction with target protein.

## 3 Results

### 3.3 Identification of the berberine therapeutic target genes against KIRC/COVID-19

**Figure 2:**
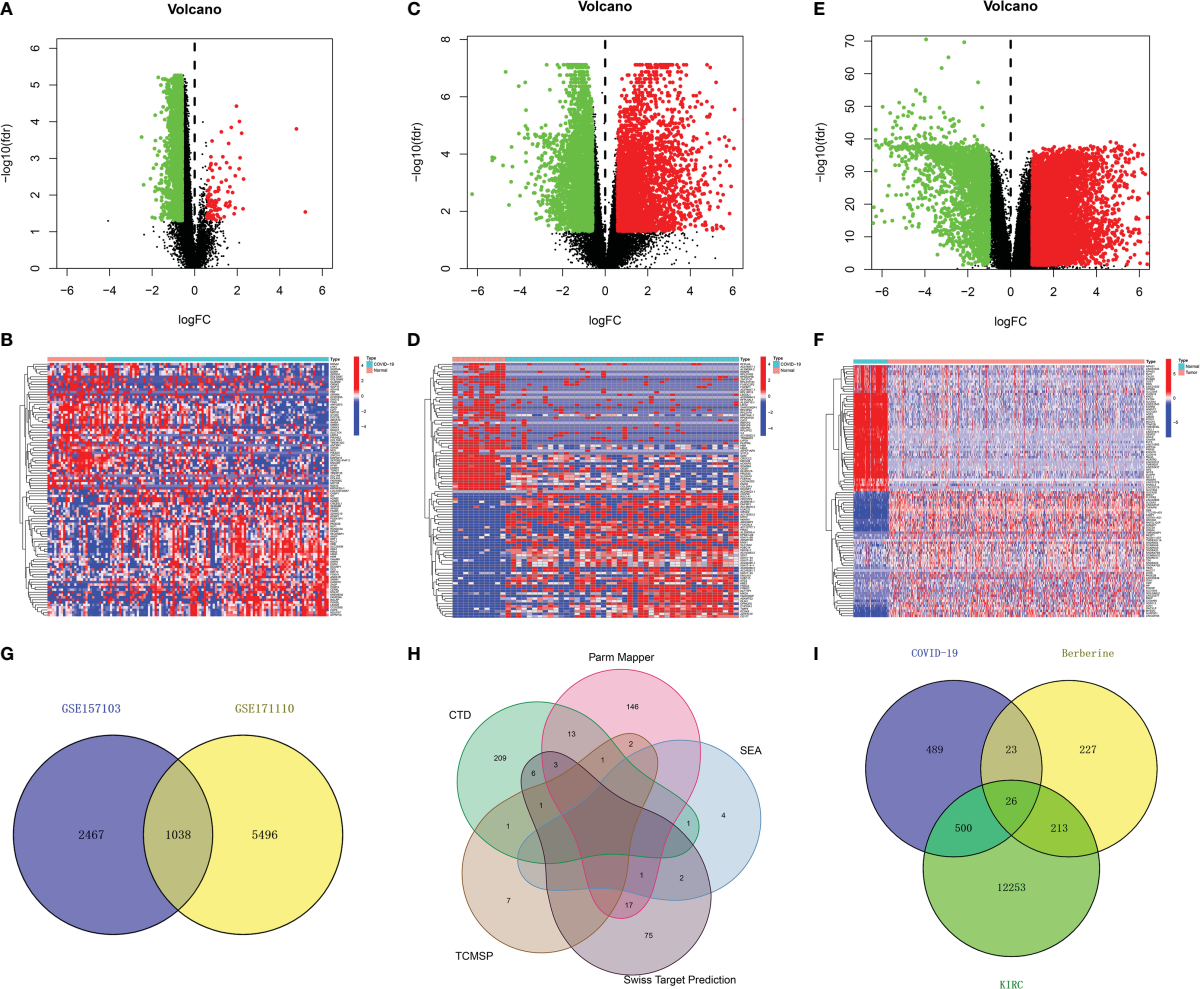
Identification of therapeutic target genes of berberine against KIRC/COVID-19. **(A, B)** Volcano plot and heatmap of DEGs from GSE157103 data set, and heatmap shown the top 50 DEGs **(C, D)** DEGs from GSE171110 data set. **(E, F)** DEGs from TCGA data set. **(G)** The intersection of GSE157103 and GSE171110 data set. **(H)** The union set of berberine target genes of each website. **(I)** The intersection of COVID-19 DEGs, KIRC DEGs and berberine target genes.

In total, 19,472 genes were obtained from 10 healthy individuals and 100 COVID-19 patients in the GSE157103 dataset, and 30,183 genes were obtained from 10 healthy individuals and 44 COVID-19 patients in the GSE171110 dataset. Following identification between the healthy and COVID-19 group based on the filtering criterion of *p* < 0.05, and | log fold change | of > 0.585, 3,505 DEGs (3,399 downregulated and 106 upregulated genes) in the GSE157103 dataset and 6,534 DEGs (3,070 downregulated and 3,464 upregulated genes) in the GSE171110 dataset were obtained (**Figures 2A–D**). To exclude interference factors and enhance credibility, DEGs in the two datasets were intersected, and 1,038 overlapping genes were considered crucial for COVID-19 infection (**Figure 2G**).

In total, 38,125 genes from 72 normal and 539 KIRC tissues were obtained from TCGA. Similarly, 12,992 KIRC DEGs (4,199 down-regulated and 8,793 upregulated genes) (**Figures 2E, F**) were obtained.

Additionally, 235, 183, 8, 100, and 12 berberine target genes were obtained from CTD, Parm Mapper, SEA, Swiss Target Prediction and TCMSP, respectively. After merging, 489 berberine target genes were obtained (**Figure 2H**).

In total, 526 DEGs were co-expressed in KIRC/COVID-19 after intersecting, and 26 genes were obtained by crossing 489 berberine target genes with 1,038 COVID-19 and 12,992 KIRC DEGs, indicating that berberine plays a therapeutic role in KIRC/COVID-19 through these genes (**Figure 2I**).

### 3.4 Exploration of the underlying therapeutic mechanism of berberine against KIRC/COVID-19

**Figure 3:**
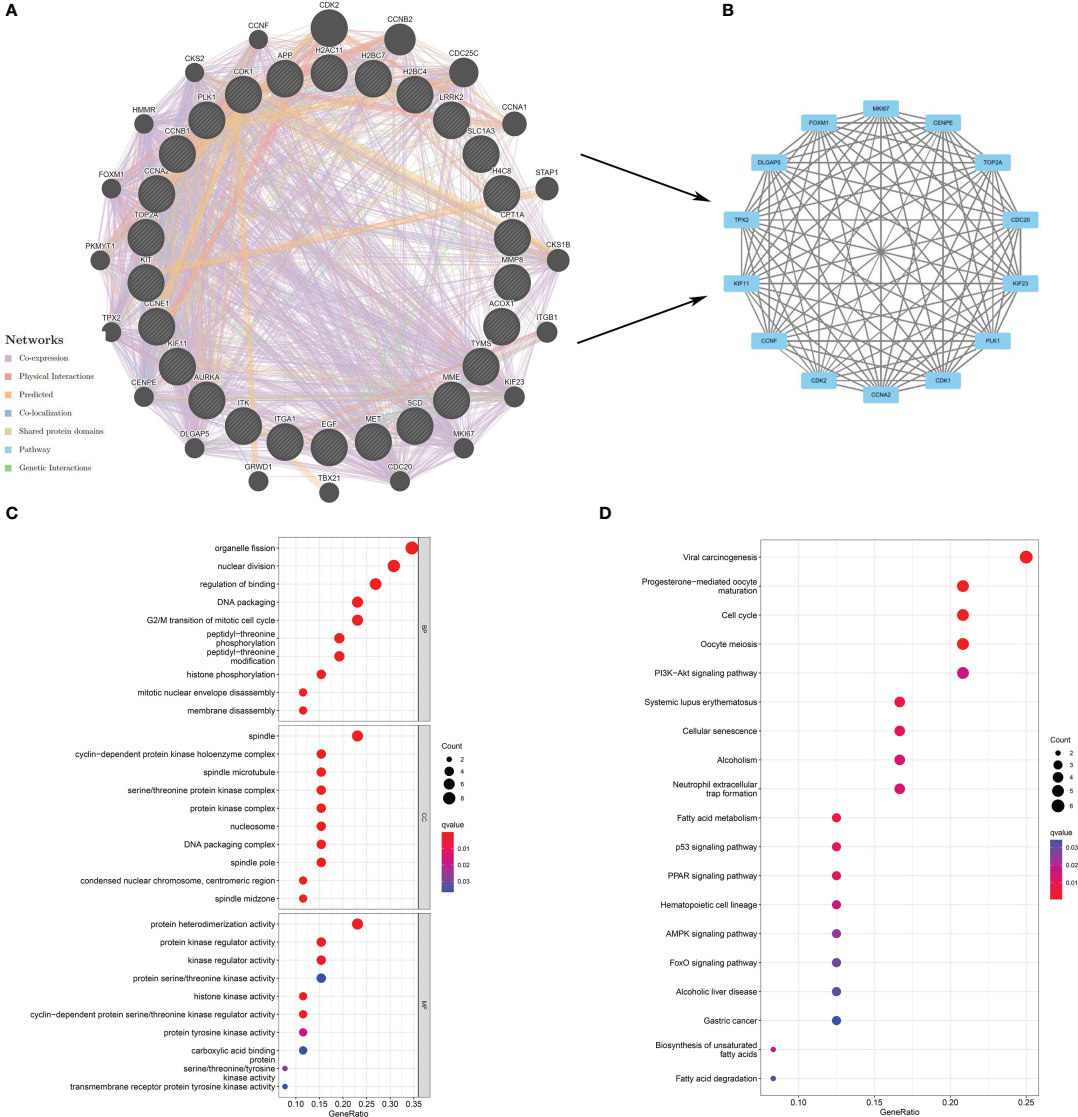
Exploration of the potential mechanism of berberine against KIRC/COVID-19. **(A)** The PPI network based on therapeutic target genes of berberine against KIRC/COVID-19. **(B)** the core network of PPI. **(C)** GO analysis of target genes. **(D)** KEGG pathway analysis of target genes.

Proteins, the result of transcription and translation of genes, are elementary substances that directly affect the metabolism, physiology, and pathology of the human body. To verify protein interactions, 26 berberine genes against KIRC/COVID-19 were imported into the GeneMANIA website, and a PPI network was constructed (**Figure 3A**). Next, the PPI network data were loaded into Cytoscape software, and the topological parameters, including Betweenness-Centrality, Closeness-Centrality and Degree, were calculated *via* the package “CytoNCA”. Following filtering with a criterion of topological parameters greater than the median, 14 predominant proteins were established in the core network (**Figure 3B**) (**Table 1**).

**Table 1:** Topological parameters of 14 preponderant proteins.

Proteins	Betweenness-Centrality	Closeness-Centrality	Degree
CDK1	71.39674	0.661765	318
CCNA2	36.55376	0.661765	305
PLK1	55.95462	0.681818	280
CDC20	129.6673	0.703125	269
KIF23	42.9122	0.671642	249
TOP2A	65.36013	0.661765	244
CENPE	73.81254	0.671642	230
FOXM1	34.29189	0.661765	226
DLGAP5	58.82625	0.652174	215
MKI67	38.57207	0.633803	213
TPX2	76.28393	0.633803	205
KIF11	50.88171	0.671642	183
CCNF	45.66335	0.642857	147
CDK2	100.7197	0.671642	104

GO analysis revealed that 26 genes were enriched in 771 GO terms, including 669 biological processes (BPs), 85 cellular components (CCs), and 17 molecular functions (MFs). The dominant BPs were organelle fission, nuclear division, binding regulation, DNA packaging, and the G2/M transition of the mitotic cell cycle (**Figure 3C**). Nineteen KEGG terms were enriched in the KEGG analysis, including viral carcinogenesis, progesterone-mediated oocyte maturation, cell cycle, oocyte meiosis, and PI3K−Akt signalling pathway (**Figure 3D**).

### 3.5 Clinical significance of target genes in COVID-19 patients

**Figure 4:**
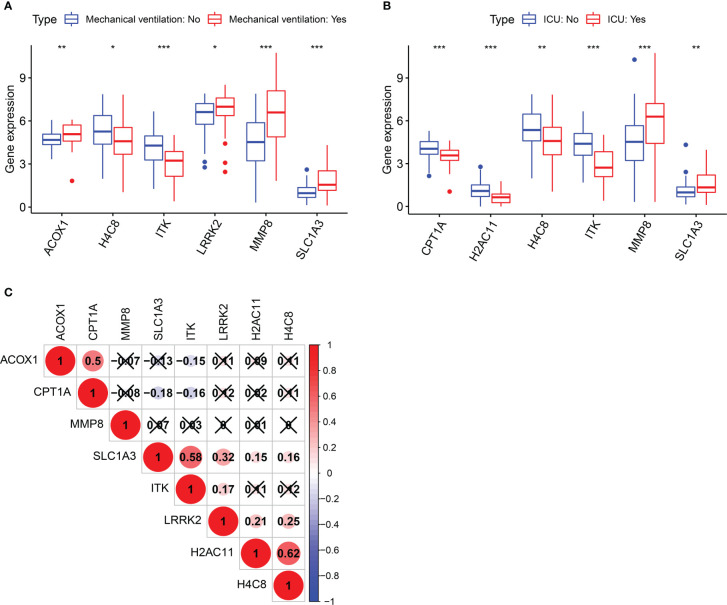
Clinical significance of target genes in COVID-19 patients. **(A)** Boxplot of target genes associated with mechanical ventilation. **(B)** Boxplot of target genes associated with ICU admission. **(C)** The correlation analysis between genes after screening. *p <0.05, **p <0.01, ***p <0.005.

Clinical information in the GSE157103 dataset includes mechanical ventilation and ICU, which are pivotal indicators for evaluating COVID-19 severity. Following the Wilcoxon test, six of the 26 berberine target genes against KIRC/COVID-19 were associated with mechanical ventilation. Compared to the non-mechanical ventilation group, the expression of ACOX1, LRRK2, MMP8, and SLC1A3 was higher, and that of H4C8 and ITK was lower in the mechanical ventilation group (**Figure 4A**). As MMP8 and SLC1A3 expression increased and CPT1A, H2AC11, H4C8, and ITK expression decreased, the probability of ICU admittance increased (**Figure 4B**). In addition, the correlation between genes was demonstrated using the Spearman correlation test (**Figure 4C**). These results revealed that berberine treatment could effectively reduce the utilisation rate of mechanical ventilation and the probability of ICU admittance by targeting these eight genes and inducing a chain reaction among them.

### 3.6 Assessment of target gene signatures in KIRC patients

**Figure 5:**
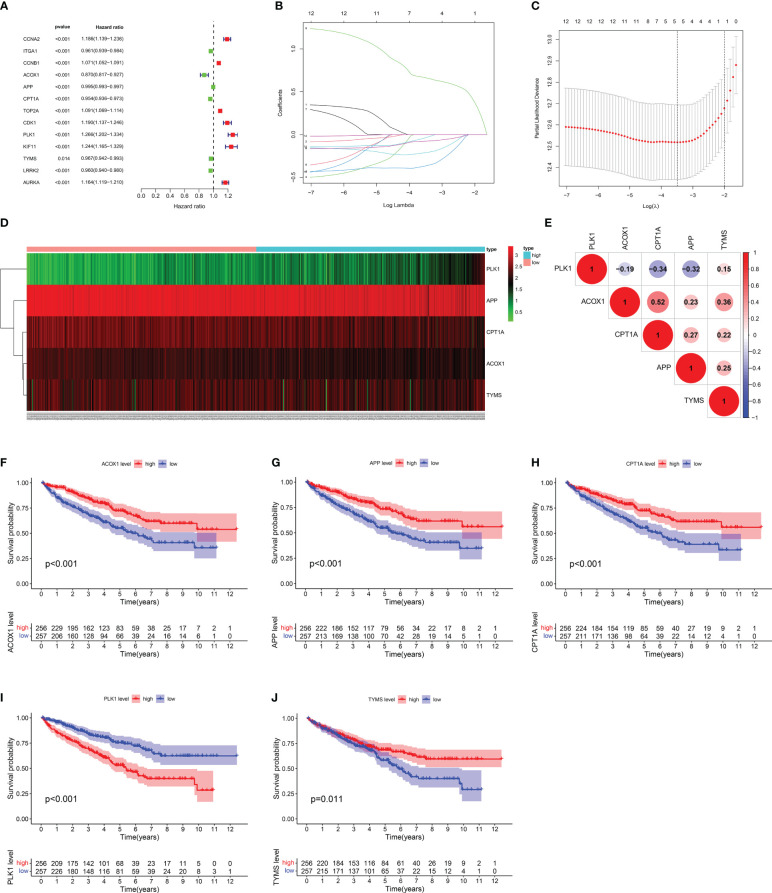
Assessment of target gene signatures for KIRC patients. **(A)** The forest plot of univariate Cox proportional analysis. **(B, C)** The LASSO Cox regression analysis for detecting the representative gene. **(D)** Heatmap of target gene signatures. **(E)** The correlation analysis between target gene signatures. **(F–J)** Kaplan-Meier survival analysis of target gene signatures.

**Figure 6:**
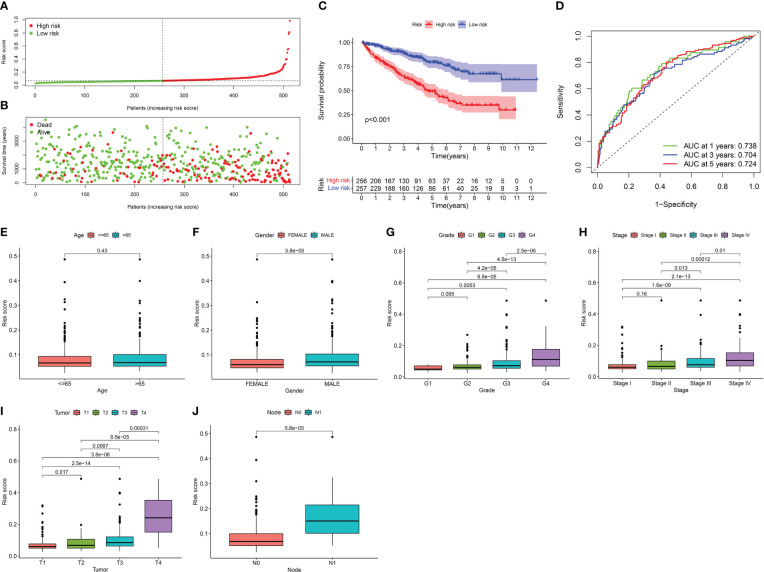
Clinical significance of risk signature for KIRC patients. **(A)** Distribution of risk scores to be divided into high- and low- risk groups. **(B)** Distribution of the OS to depict the relationship between OS and risk signature in dead and alive KIRC patients. **(C)** Kaplan-Meier survival analysis of risk signature. **(D)** AUC in ROC analysis for risk signature at 1‐, 3‐and 5‐years survival. When AUC is greater than 0.7, the prediction model has reliable accuracy. **(E–J)** Boxplot reveals the relationship between risk signature and clinical information, containing in age, grade, tumor stage, tumor, lymph node and metastasis deteriorated.

According to univariate Cox regression analysis, 13 of the 26 berberine target genes against KIRC/COVID-19 were closely correlated with OS (**Figure 5A**). To reduce the influence of multicollinearity among variables and detect the optimal genes, LASSO Cox regression analysis was performed; ACOX1, APP, CPT1A, PLK1 and TYMS were identified as representative signatures of the OS of KIRC patients, and the risk score was forecasted by the computational formula: risk score = (-0.365 × expression ACOX1) + (-0.211 × expression APP) + (-0.077 × expression CPT1A) + (0.663 × expression PLK1) + (-0.036 × expression TYMS) (**Figures 5B, C**). According to their number, KIRC patients were subdivided into high- and low-risk groups (**Figure 6A**). The heatmap vividly described the expression of five genes in these groups (**Figure 5D**). Furthermore, these five genes correlated positively or negatively (**Figure 5E**). Kaplan-Meier (KM) survival analysis revealed that high-expression genes, including ACOX1, APP, CPT1A, and TYMS, had a higher survival probability over time, whereas PLK1 had the opposite effect (**Figures 5F–J**).

### 3.7 Clinical significance of risk signature in KIRC patients

The OS of KIRC patients decreased with an increased risk score, and the survival rate tapered towards death (**Figure 6B**). KM survival analysis revealed that the prognosis of the high-risk group was substantially worse than that of the low-risk group (**Figure 6C**). The AUC, a sensitive and specific risk signature indicator, showed reliable predictive accuracy at the 1-, 3-, and 5‐year survival time points (**Figure 6D**). ​Except for age, significant differences were observed in the risk signature for each clinical feature. The risk signature increased to varying degrees as the grade, tumour stage, tumour, lymph node, and metastasis deteriorated (**Figures 6E–J**). These results suggest that the risk signature can effectively reflect the clinical status and prognosis of KIRC patients.

### 3.8 The immune response of target genes for KIRC/COVID-19 patients

**Figure 7:**
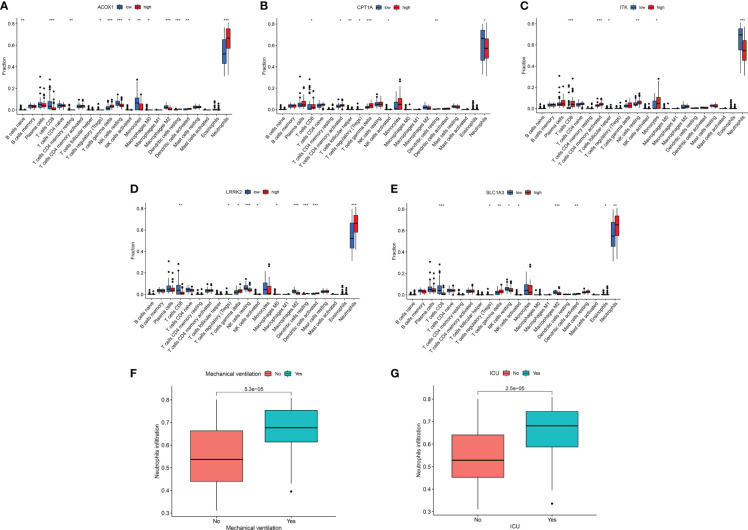
Immune response of target genes for COVID-19 patients. **(A–E)** Boxplot of target genes associated with 22 immune cell infiltration. **(F)** Boxplot of neutrophil infiltration associated with mechanical ventilation. **(G)** Boxplot of neutrophil infiltration associated with ICU admission. *p <0.05, **p <0.01, ***p <0.005.

In total, 22 immune cell infiltration was predicted using “CIBERSORT”. We focused on the comparatively large number of neutrophil infiltrations in COVID-19 patients, possibly associated with a sharp neutrophil increase and activation of SARS-CoV-2-induced neutrophil extracellular traps (41–44) (**Figures 7A–F**, **Supplementary File 1**). According to the boxplot, high expression of ACOX1, LRRK2, and SLC1A3 was susceptible to neutrophil infiltration, whereas that of CPT1A and ITK was resistant to neutrophil infiltration (**Figures 7A–E**). The relationship between these genes and neutrophil infiltration was consistent with the relationship between the genes and the utilisation rate of mechanical ventilation or probability of ICU admittance (**Figures 4A, B**). Moreover, high neutrophil infiltration significantly affected the increased use of mechanical ventilation and ICU stay (**Figures 7F, G**).

**Figure 8:**
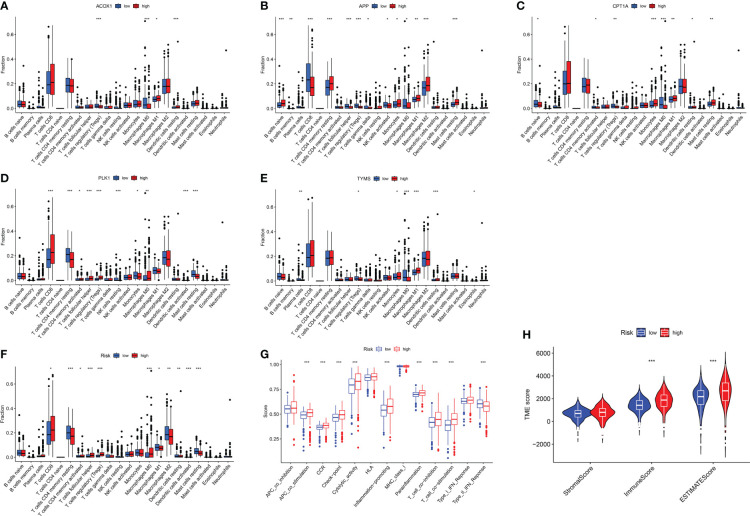
Immune response of target genes for KIRC patients. **(A–F)** Boxplot of target gene signatures associated with 22 immune cell infiltration. **(G)** The analysis of immunological functions. **(H)** the tumor microenvironment score. *p <0.05, **p <0.01, ***p <0.005.

In KIRC patients, five genes were positively or negatively correlated with 22 immune cells to varying degrees, indicating that berberine may play a therapeutic role by regulating the infiltration of immune cells (**Figures 8A–E**). Furthermore, seven immune cells, including CD8 T cells, CD4 memory-activated T cells, follicular helper T cells, regulatory T cells (Tregs), macrophages M0, macrophages M1 and activated dendritic cells, were enriched with an increased risk signature; however, CD4 memory resting T cells, macrophages M2, dendritic cells, and resting mast cells, were reduced (**Figure 8F**). Analysis of immunological function demonstrated that the high-risk group exhibited enhanced APC co-stimulation, CCR, checkpoint, cytolytic activity, inflammation-promotion, parainflammation, T-cell co-inhibition, and T-cell co-stimulation, and inhibited type II IFN response (**Figure 8G**). ​The violin plot showed that the high-risk groups correlated with a higher immune score, suggesting that high-risk groups are prone to a hyperimmune response (**Figure 8H**).

### 3.9 Molecular docking of berberine binding to KIRC/COVID-19 target genes

**Figure 9:**
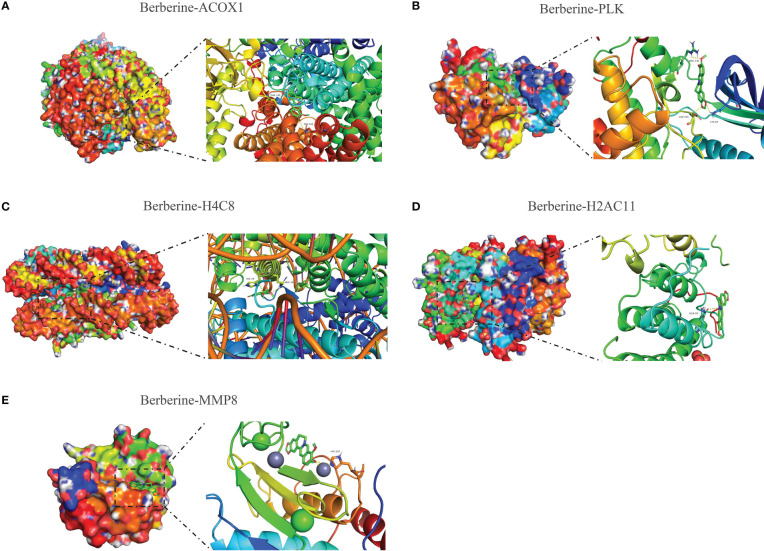
Molecular docking of berberine and target genes. The top 5 docking affinity score. **(A)** Berberine binding to ACOX1. **(B)** Berberine binding to PLK. **(C)** Berberine binding to H4C8. **(D)** Berberine binding to H2AC11. **(E)** Berberine binding to MMP8.

Molecular docking was performed to determine the optimal binding mode of berberine to KIRC/COVID-19 target genes. The docking affinity score output by AutoDock Vina was ACOX1 (-10.4) < PLK1 (-10.1) < H4C8 (-9.7) < H2AC11 (-7.9) < MMP8 (-7.9) < LRRK2 (-7.8) < SLC1A3 (-7.8) < ITK (-7.7) < TYMS (-7.1) < APP (-6.1) < CPT1A (-5.5). All docking affinity score was < -5.0 kcal/mol, indicating that these target genes play an important role in the berberine treatment of KIRC/COVID-19 (**Figures 9A–E**, **Supplementary File 2**).

## 4 Discussion

The COVID-19 epidemic has significantly affected individuals worldwide and had catastrophic effects on the global economy, health, and psychology (45). ​ Although the vaccine received immediate popularity, the anti-COVID-19 vaccine campaign persisted for years because of the dreadful contagiousness, SARS-CoV-2 mutations, and declining antibody titers (3–6). Although specific medicines have not yet been developed, COVID-19 therapy schedules and management strategies have been continuously investigated (46–48). In China, the mortality rate of COVID-19 patients is 0.41% (24,232 deaths/59,69737 confirmed cases), which is significantly lower than the global average, indicating the superior effectiveness of conventional Chinese medicine in combating COVID-19 (49, 50), such as Lianhua Qingwen capsules, Xuanfei Baidu decoction, and Xuebijing, among others (51). SARS-CoV-2 invades the kidney, harms renal cells, and impairs renal function (52–54), posing a greater threat to KIRC patients. Additionally, KIRC patients are more likely to contract COVID-19 and are more susceptible to dying from its infection (8, 9, 19). We sought an effective therapeutic strategy to decrease possible significant sequelae and increase the survival of KIRC/COVID-19 patients based on the relationship between COVID-19 and KIRC. It has been demonstrated that berberine, an active ingredient in most natural herbs, lowers inflammation in COVID-19 patients and destroys KIRC cells. Using computer-based approaches and algorithms, the possible therapeutic mechanism of berberine in treating KIRC/COVID-19 patients was investigated.

In this study, 26 genes were identified as therapeutic target genes of berberine against KIRC/COVID-19. The PPI network produced by the GeneMANIA website revealed that the 26 therapeutic target genes achieved protein function through a core network composed of 14 genes, such as CDK1, CCNA2, and PLK1, among others. GO analysis revealed that the BPs of 26 target genes were enriched mainly in organelle fission, nuclear division, binding regulation, DNA packaging, and G2/M transition of the mitotic cell cycle. These BPs play an important role in cell proliferation, which is crucial in KIRC/COVID-19 pathogenesis (55, 56). The PI3K-Akt signalling pathway predicted by KEGG analysis was activated by accelerating the phosphorylation of Akt, mTOR, 4E-BP1, and S6K1 during the initial phases of COVID-19 infection (57), resulting in the rapid activation of the translation machinery of viral protein synthesis (58, 59). The MDM2 protein is activated by the aberrant PI3KAkt signalling pathway in KIRC, allowing easier degradation of the tumour suppressor p53 by proteasome machinery (60). According to functional analyses, the berberine mode of action in treating KIRC/COVID-19 may have diverse pathways that interfere with tumour cell proliferation or viral self-replication, particularly the PI3K-Akt signalling pathway.

Mechanical ventilation and ICU admittance are indispensable life support measures for patients with severe COVID-19 (61–63). ​The severity of COVID-19 infection in patients intensified as the expression of ACOX1, LRRK2, MMP8, and SLC1A3 increased, and the expression of CPT1A, H2AC11, H4C8, and ITK decreased. These genes play different roles in resisting viral infections and can function as prospective targets for COVID-19 treatment. The enhanced MMP8 production during the early stages of COVID-19 infection is directly linked to the breakdown of the extracellular matrix (ECM), including collagen, laminin, and proteoglycans, which promotes viral multiplication and inflammation (64). ​ROS production, which is essential for inflammation and lung injury, is enhanced by high levels of ACOX1 protein and function (65, 66). Huang et al. found that IL-2-inducible T-cell kinase (ITK) facilitates γδ T-cell-derived IL-17A production to resist *Mycobacterium tuberculosis* infection (67). By inhibiting CPT1a expression, increased mitochondrial ROS accelerates the progression of pulmonary fibrosis; however, restoring CPT1a expression impedes pulmonary fibrosis (68). ​ These genes function by controlling ROS production or being controlled by ROS, which may be connected to the interference of berberine with ROS production. Thus, berberine decreases ROS production by controlling the expression levels of these genes, lowering mechanical ventilation and ICU admittance in COVID-19 patients.

Five of the 26 target genes of berberine against KIRC/COVID-19 were closely associated with OS in KIRC patients, as determined by univariate and LASSO Cox regression analyses., Du et al. discovered that lipid deposition plays a significant role in accelerating KIRC progression with hypoxia-inducible factors (HIFs)-induced CPT1A suppression, and with the reversing of this inhibition, CPT1A decreases KIRC progression (69). Increasing fatty acid oxidation by enhancing CPT1A expression may be a promising therapeutic strategy for KIRC (70). Furthermore, HIF-2 increased PLK1 expression, inducing metastasis and drug resistance in KIRC cells (71) and counteracting CPT1A effects. By phosphorylating MCM3, PLK1 promoted KIRC cell growth and prevented apoptosis (72). Regulation of the antagonistic effect between CPT1A and PLK1 may be an advancement to improve the OS of KIRC patients. The survival status of KIRC patients may be more thoroughly and objectively reflected by the risk signature derived from the five gene expression levels in this study and may accurately reveal the change mechanism of tumour grade, stage, lymph nodes, and metastasis. All of the evidence demonstrates the risk signature as a valid indicator of patient prognosis and a tool for evaluating the effectiveness of KIRC.

The immune response significantly contributes to disease development. The fulminant immune response is a body characteristic following SARS-CoV-2 infection (73–75). In this study, abundant neutrophil infiltration was observed in COVID-19 patients compared with the remaining 21 immune cells. This is similar to the results of several studies, and the sharp increase in neutrophils and activation of neutrophil extracellular traps caused further acute injury to the lungs and kidneys (41–44, 76). Cytokine storm-induced inflammation activates ROS, causing severe complications (77). Therefore, it is practical to reduce mechanical ventilation and ICU admittance by decreasing neutrophil infiltration. Abnormal immunological responses in KIRC patients are a significant contributor to disease onset. KIRC patients with a high immune score were at increased risk, suggesting KIRC progressed when a significant proportion of immune cells were concentrated in the tumour microenvironment (TME). In recent years, immune checkpoint inhibitors have gradually become a mainstream therapy and research focus for KIRC (78) and are beneficial for improving the prognosis of patients (79–81). Relative to chemotherapy, treatment with immune checkpoint inhibitors does not appear to significantly increase risk of serious adverse events in COVID-19-positive patients (82). Correcting immune disorders may be a future trend for KIRC treatment, immune checkpoint inhibitors remains an important treatment option even in COVID-19 positive patients.

The treatment mechanism of berberine against KIRC/COVID-19 remains uncertain. In this study, DEGs were screened, and target genes for correlation with clinical information were analysed, revealing immune mechanisms. Finally, molecular docking was used to investigate the best binding mode of berberine to the KIRC/COVID-19 target genes. The results indicate that berberine treatment is an effective strategy for treating KIRC/COVID-19. In a follow-up study, COVID-19-infected KIRC cells will be examined using berberine, and the role of target genes in the treatment process will be analysed to further explore the therapeutic mechanism of berberine against KIRC/COVID-19.

Overall, KIRC patients are at higher risk of contracting COVID-19, and no effective therapies have been developed for both conditions. In this study, berberine was demonstrated as a potential treatment option in pharmacological, immunological, and clinical practice; its therapeutic effect provides a potential and reliable treatment option for patients with KIRC/COVID-19.

## Data availability statement

Publicly available datasets were analyzed in this study. This data can be found here: https://www.ncbi.nlm.nih.gov/geo/, https://portal.gdc.cancer.gov/repository, http://ctdbase.org/ http://www.lilab-ecust.cn/pharmmapper/, https://sea.bkslab.org/, http://www.swisstargetprediction.ch/, http://tcmspw.com/tcmsp.php/.

## Author contributions

ZZ, KZ and YZ developed the concept of the project. The data was collected by ZZ, WL and FY, and evaluated by XL, KN and XW. KZ and YZ conducted a repeat experiment.All authors reviewed and discussed the results and contributed to the paper preparation. ZZ, KZ and YZ wrote the manuscript. All authors contributed to the article and approved the submitted version.

## Supplementary material

Supplementary Figure 1 Boxplot of H2AC11, H4C8 and MMP8 associated with 22 immune cell infiltration. Without statistical significance in neutrophil infiltration. **p <*0.05, ***p <*0.01, ****p <*0.005.

Supplementary Figure 2 Molecular docking of berberine and target genes.
