# Supplementary figures and images for: Identification of berberine as a potential therapeutic strategy for kidney clear cell carcinoma and COVID-19 based on analysis of large-scale datasets
Source: Front Immunol. 2023 Mar 23;14:1038651. doi: 10.3389/fimmu.2023.1038651 (PMC10076552; doi:10.3389/fimmu.2023.1038651)

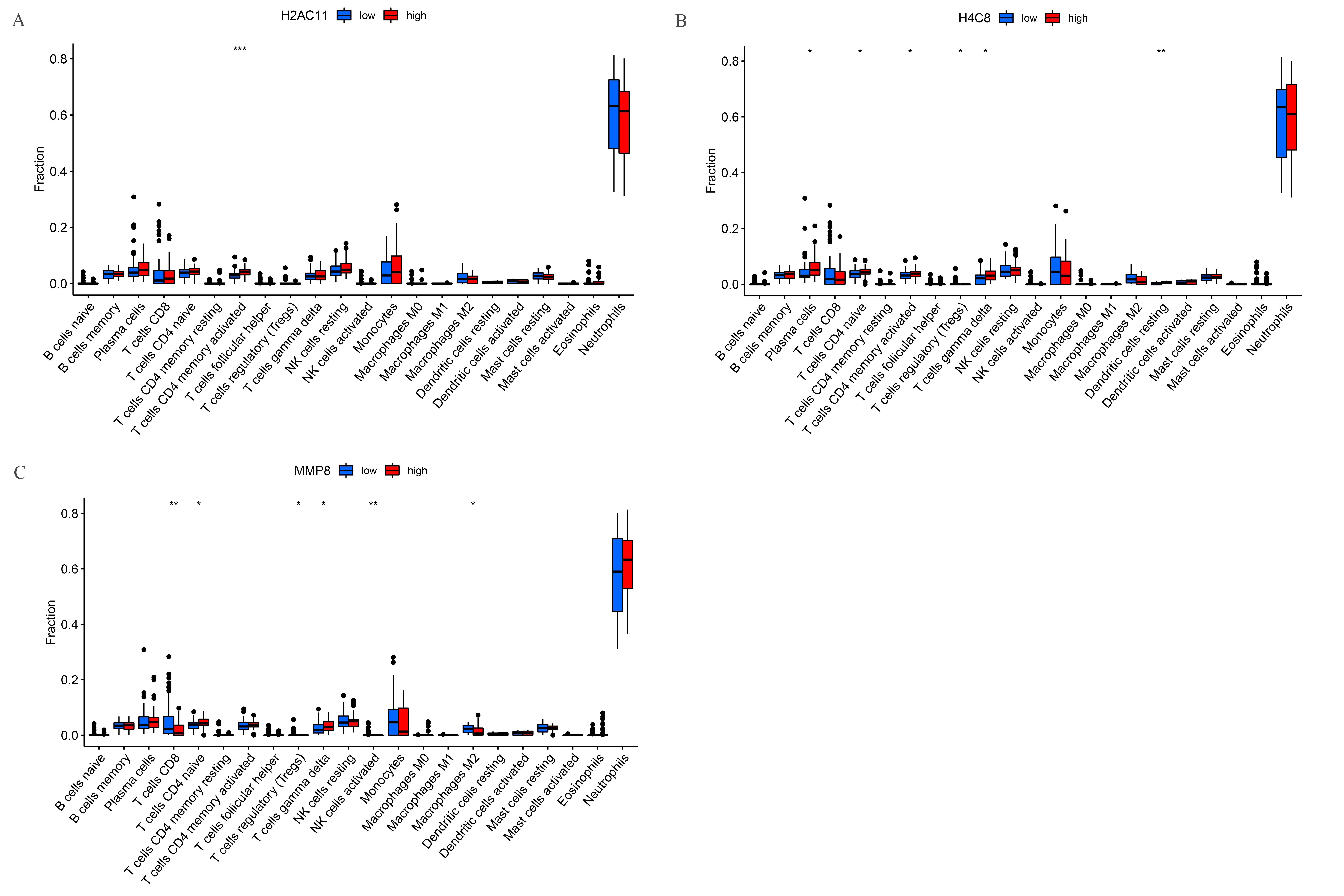

Supplement: Supplementary Figure 1 — Boxplot of H2AC11, H4C8 and MMP8 associated with 22 immune cell infiltration. Without statistical significance in neutrophil infiltration. *p <0.05, **p <0.01, ***p <0.005. [file Image_1.jpeg]
